# Insulin Growth Factor-I in Protein-Energy Malnutrition during Rehabilitation in Two Nutritional Rehabilitation Centres in Burkina Faso

**DOI:** 10.1155/2009/832589

**Published:** 2009-03-17

**Authors:** S. Kouanda, B. Doulougou, V. De Coninck, L. Habimana, B. Sondo, R. Tonglet, J. M. Ketelslegers, A. Robert

**Affiliations:** ^1^Institut de recherche en Sciences de la Santé, Centre national de la recherche Scientifique et Technologique, Ministère des Enseignements Secondaire, Supérieur et de la Recherché Scientifique, BP 7192 Ouagadougou, Burkina Faso; ^2^Unité de Diabétologie et de Nutrition, Avenue Hippocrate 54, Bte 5474, 1200 Woluwe Saint Lambert, Brussels, Belgium; ^3^Unité d'épidémiologie, biostatistique et méthodes opérationnelles en santé publique, Clos Chapelle aux Champs, 30, bte 3058, 1200 Woluwe-saint-Lambert, Brussels, Belgium

## Abstract

*Objective*. To investigate the relationship between IGF-I and the nutritional status of West-African children hospitalised for nutritional rehabilitation. *Patients and methods*. A cohort study was performed in two centres for nutritional rehabilitation and education (CREN) in Burkina Faso. Children were followed and the anthropometric data as well as the capillary blood samples were taken on the 7th and on the 14th days after their admission. IGF-I levels were determined from dried blood spots on filter paper on IGF-I RIA, after separation of the IGF-I from its binding proteins, using Sep-Pak chromatography. *Results*. A total of 59 children was included in the cohort. The IGF-I mean geometric values (SD) were 6.3 (1.4) *μ*g/L on admission, 8.6 (1.8) *μ*g/L at day 7 and 13.6 (2.0) *μ*g/L at day 14. The differences between these values were statistically significant (*P* < .001). There is a significant correlation between the changes of IGF-I with the change of weight for height Z-score (*P* = .01). *Conclusion*. These results suggest that IGF-I can be considered as a potential marker to follow the nutritional status of children admitted in hospital for protein and energy malnutrition.

## 1. Introduction

Protein-energy malnutrition (PEM) remains a major public health problem in the world, particularly in developing countries. According to the World Health Organization (WHO), PEM affects one-third of children around the world, and 43% of children in developing countries (230 million), presenting a delay in staturoponderal growth [1]. In Burkina Faso, the prevalence of children with acute malnutrition is 19%, while 39% present a delay of statural growth, and 38% have weight insufficiency [2].

The usual signs of children malnutrition are clinical signs and anthropometric measures. However, biological markers such as albumin, transthyretin, transferrin, and retinol binding protein are also used for nutritional diagnosis and rehabilitation aftercare. Several surveys have shown the relationship between clinical, anthropometric, or biological indicators and mortality of children suffering from PEM in hospitals [3–5].

More recently used markers like insulin-growth factor-I (IGF-I) used to measure the deficit in growth hormone (GH), and the insulin growth factors binding proteins (IGFBPs) also draw attention. Insulin-like growth factor-I (IGF-I) is single-chain peptides of 7.5 kilodaltons (kDas). Her structure is similar to the IGF-II and proinsulin [6, 7]. The liver is believed to be the main source of production of IGF-I [8–10], but the highest concentrations of IGF-I are observed in blood [11, 12]. IGF-I is also synthesized by several tissues [13, 14].

Many factors intervene in the regulation of the IGF-I but the most important are growth hormone (GH), insulin, and nutritional status [15].

Nutritional status plays an important role in the regulation of the IGF-I. Adequate food intake is essential for maintaining normal IGF-I and IGFBP-3 circulating rates in the serum[16, 17]. Indeed, energy and protein restrictions in children lead to a decrease of circulating IGF-I and IGFBP-3 rates [18].

The variations of serum IGF-I rates observed in response to different nutritional states suggest that IGF-I may serve as a marker for children nutritional state [19–21]. The IGF-I appears indeed as a marker potentially more sensitive than the serum albumin or the transferrin, even if the effect of infection on circulating concentration must be taken into account [22–24].

Only two surveys have been conducted on IGF-I and the nutritional rehabilitation among children under 5 years old but both were inconclusive because of low sample sizes [22, 24], but both were inconclusive because of low-sample sizes. These two studies did not also analyse the change of IGF-I and the changes of nutritional status. So, the purpose of our study is to analyse the relationship between the IGF-I values and the nutritional status of children hospitalised for nutritional rehabilitation in the context of a West-African country.

## 2. Patients and Methods

The study of protocol was reviewed and approved by the Ethics Committee for Research in Health of Burkina Faso.

### 2.1. Subjects

This study was conducted in two nutritional rehabilitation centres: (CREN) of the regional hospital (CHR) and the Persis medical centre (CM) of the city of Ouahigouya, located in the northern part of Burkina Faso.

All consecutively admitted patients with PEM should be included in the study. Among these patients, the criteria for selection were the age <60 months and the absence of known pathology like HIV, diabetes, congenital diseases, and tumours.

Upon admission, social and demographic characteristics (age, sex, and vaccination past records) were collected; weight and height were measured, and different clinical data were checked (presence of oedema, coloration of hair, splenomegaly, and hepatomegaly). Then, a capillary blood sample was taken from the child finger. The children were followed up and the anthropometric data and the capillary blood samples were again taken again on the 7th and the 14th days after admission.

The children admitted for malnutrition were well-cared for. They benefited from a free standard feeding which consisted of milk F75 for 7 days at 135 Kcal/Kg weight per day and milk F100 after the week at 100 Kcal/Kg weight per day. This posology was increased by 20 Kcal every 3 days until 200 Kcal/Kg per day, as necessary. In addition, the children received enriched pulp five times a day. A treatment for bacterial infections and malaria was available at any time if required.

A total of 68 children were recruited for the study but only 59 were included because the ages of 9 children were unknown. Table 1 shows the sociodemographic characteristics of the 59 children. The sex ratio was 1.68 for boys; 76.3% of the children were aged between 1 and 2 years. Most of the mothers (96.6%) and the fathers (88.1%) had no scholarship education. Mothers were mainly housewives and fathers were mainly farmers. With regard to vaccine status, two thirds of the children were fully vaccinated, 30.9% not fully vaccinated, and 3.6% were not vaccinated at all. The average maternal breast-feeding duration was 9 ± 3 months.

### 2.2. Evaluation of Nutritional Status

Weight and height were determined on a Salter balance and the height was rounded to the nearest centimetre. For children younger than 2 years, height (length) was measured on a supine table as follows: an assistant held the head against the headboard and another straightened the legs. For children older than 2 years, a stadiometer was used. Nutritional status, expressed as weight for height (WHZ) and height for age (HAZ), was standardised for age and sex using either the reference tables from the Burkina Faso reference tables [25] or the United States National Center for Health Statistics (NCHS) [26, 27]. The reference tables of Burkinabe children were constructed from anthropometric measures collected during the survey on the reference values of IGF-I in Burkina Faso [25].

### 2.3. Blood Sampling

Blood was obtained by vein puncture and 2 drops per circle were immediately collected on (free falling) the filter paper; 2 circles per paper were systematically filled; the samples were dried after 5–10 minutes at ambient temperature (30–35°C) and were kept at 4°C for 1–2 weeks before processing.

All filter paper samples were transferred for analyses to the Unit of Diabetes and Nutrition, Belgium. Samples were collected between October 2005 and May 2006.

### 2.4. Sample Processing and IGF-I Assay

We validated the methods of determining the IGF-I levels from dried blood spots on filter paper on IGF-I RIA, after separating the IGF-I from its binding proteins using Sep-Pack chromatography [25].

The extraction recovery of IGF-I was of 84.4 ± 3.4%. IGF-I was determined by RIA, using the antiserum at a 1/15000 dilution, and ^125^I- IGF-I was labeled by the iodogene method and purified by RP-HPLC. The concentration of the standard curve spans over a range of 0.05 to 2 ng/mL. Intra-assay and total imprecision of the RIA were of 5% and 16% at a level of 0,25 ng/mL and of 5% and 22% at a level of 1.01 ng/mL [25].

IGF-I absolute measurements were also standardised for age and sex, and reported as IGF-I Z scores, using the reference values of IGF-I in children from birth to the age of 5 in Burkina Faso [25].

### 2.5. Statistical Methods

Results are expressed as proportions for discrete variables, as means and standard deviations (SDs) for continuous variables with a normal distribution, and as geometric means with geometric standard deviations for continuous variables with a log-normal distribution, like IGF-I. Standardised values are expressed as Z-scores and are reported as mean ± SD and proportion under −2.00 or under −3.00. IGF-I was considered either as an absolute measurement and analysed on a log scale, or as an age- and sex-adjusted normal Z-score and analysed on a linear scale. Relationships between IGF-I and WHZ at baseline and after 14 days of nutritional rehabilitation were assessed using *r*, the Pearson cross-product correlation coefficient. Changes in IGF-I after 14 days of nutritional rehabilitation were expressed as ratio for absolute measurements and as differences for standardised values, and changes were tested using Student's paired *t*-test. Relationships between changes in IGF-I and increases in WHZ were assessed using also *r*. No *P*-value was corrected for multiple comparisons and a *P* value <.05 was considered as statistically significant. Statistical analyses were performed using SPSS 15.0 software.

## 3. Results

### 3.1. Evaluation of the Nutritional Status and IGF-I Values of Children upon Admission and during Rehabilitation

Two children (3.4%) were admitted to the Nutritional Rehabilitation Centre because of kwashiorkor, 55 children (93.2%) were admitted for marasmus, and two presented with both kwashiorkor and marasmus. One child died during the follow-up.

At the time of admission, using international reference values as standards, 57 children (96.6%) had an age- and sex-adjusted weight for height Z-score lower than −2.0, including 43 children with a WHZ <−3.0. Three children presented oedemas at admission and their WHZ was lower than −2.0.

Out of the 57 children with a WHZ <−2.0, 51 (89.5%) had also an age- and sex- adjusted IGF-I Z-score of <−2.0. An IGF-I Z score of <−2.0 was also observed in 90.7 % (39/43) of children presenting with a WHZ <−3.0.

When using Burkina Faso age and sex reference values for standardising, 44% (26/59) of children had a WHZ <−2.0, and 8% (5/59) had a WHZ <−3.0. The proportion of IGF-I Z-score of <−2.0 was 89% (23/26) in children with WHZ <−2.0 and 80% (4/5) in children with WHZ <−3.0.

**Figure 1:**
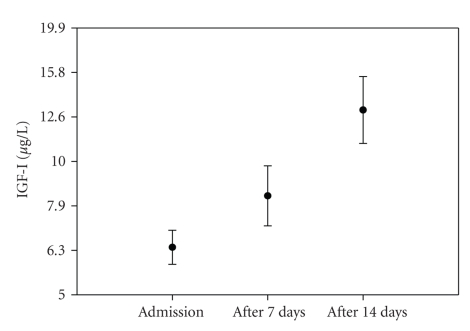
Increase in IGF-I during nutritional rehabilitation in 59 children admitted for malnutrition in Burkina Faso.

After 14 days of nutritional rehabilitation, 22 children (37%) reached a WHZ ≥−2.0 according to international references, and 91% of these children (20/22) also attained an IGF-I Z-score ≥−2.0. Using Burkina Faso data as standards, the WHZ was ≥−2.0 in 80% (*n* = 47) of children after 14 days of nutritional rehabilitation, and 81% of these children (*n* = 38) also reached an IGF-I Z-score ≥−2.0, suggesting a quick recovery of IGF-I, as illustrated in Figure 1.

During nutritional rehabilitation, IGF-I increased from 6.36 (1.40) *μ*g/L (geometric mean and SD) upon admission, to 8.59 (1.81) *μ*g/L after one week, and to 13.65 (2.0) *μ*g/L after two weeks of nutritional rehabilitation (Figure 1).

Table 2 shows the parallel increase in weight and in IGF-I, from admission to 7 and 14 days after nutritional rehabilitation. Data are presented as absolute measurements, as standardised measurements using international references for age and sex adjustments, and as standardised measurements using Burkina Faso data as standards.

After 7 days of nutritional rehabilitation, IGF-I was multiplied by 1.587, as a significant increase of 58.7% on average. After 14 days, IGF-I increase was about 151.8% on average.

There was a significant increase in log-scaled IGF-I, as well as in IGF-I Z-scores one week after admission (paired *t*-test, *P* = .003, and *P* = .007), and two weeks after admission (paired *t*-test, *P* < .001, and *P* = .001). A significant increase was also observed for weight for height Z-scores, with both standardisations.

**Figure 2:**
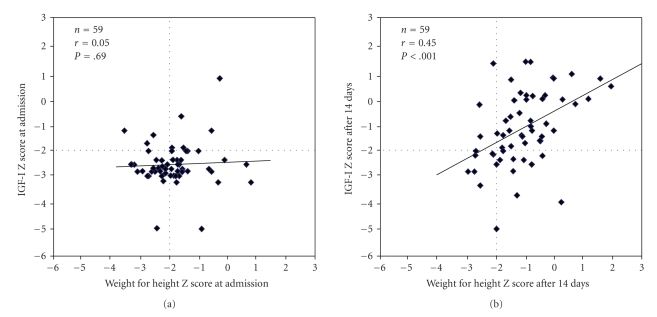
Relationship between age- and sex-adjusted IGF-I Z-score and weight for height Z-score with Burkina Faso data as references, at admission and after 14 days of nutritional rehabilitation in 59-malnourished children from Burkina Faso.

The relationship between age- and sex-adjusted IGF-I score and weight for height Z-score using the Burkina Faso reference for standardisation is illustrated in Figure 2, upon admission (a) and after 14 days (b).

Upon admission, the proportion of children who had both WHZ of <−2.0 and IGF-I Z score <−2.0 was 39% (23/59), but 47% (28/59)—a high proportion of children—had an IGF-I Z score of <−2.0 and WHZ of ≥−2.0, leading to a low correlation (*P* = .69). After 14 days of nutritional rehabilitation, the proportion of children who both had WHZ ≥−2.0 and IGF-I Z score of ≥−2.0 increased 64% (38/59), with highly significant correlation, as reflected in Figure 2(b).

**Figure 3:**
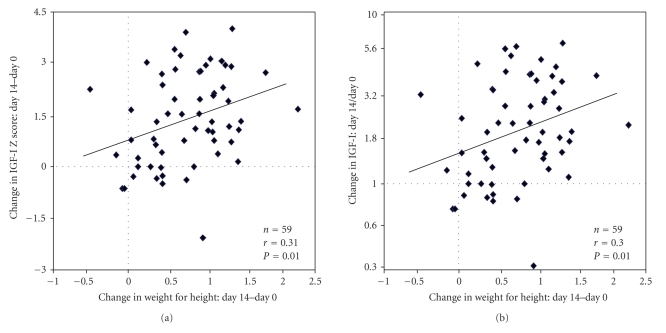
Relationship between the change in IGF-I expressed as an increase in Z-score (a) or as a ratio in absolute measurements (b) and the change in age- and sex-adjusted weight for height Z-score using Burkina Faso data as references, after 14 days of nutritional rehabilitation.

Expressing the change in IGF-I as a difference in IGF-I Z scores or expressing the change in IGF-I as a ratio between IGF-I value after 14 days and IGF-I value upon admission, there was significant correlation with the increase in weight for height Z-scores (*P* < .01 on Figure 3(a), and *P* < .01 on Figure 3(b), resp.).

When using the international NCHS references for standardisation, there was an even higher correlation between changes in IGF-I and increases in weight for height Z-scores, with *r* = 0.38 (*P* < .003) for differences in IGF-I Z scores, and *r* = 0.39 (*P* < .003) for IGF-I absolute value ratios.

## 4. Discussion

The objective of this study was to assess the relationship between the IGF-I values and the nutritional status of children hospitalised for nutritional rehabilitation. The study also sought to determine IGF-I values as forecast markers of mortality. However, this last objective has not been achieved because the care given to children admitted in rehabilitation helped maintain a low mortality rate (1 in 68 children enrolled in the present study).

One of the limitations of our study is that the influence of factors like infection was not taken in consideration. The acute or chronic inflammatory status could interfere on the rate of IGF-I production. It was shown that in the experimental model, the stimulation of an inflammatory status by injection of endotoxin leads to a reduction of IGF-I rates due to a resistance of the GH [20]. During the sepsis, Yumet et al. observed an increase of GH with a reduction of 40–50% of plasma IGF-I in rat at 12 hours and 24 hours after GH administration [28].

As far as the evaluation of nutritional status is concerned, most of our patients suffered from protein and energy malnutrition, with a few cases of kwashiorkor. Using NCHS references, only two children had a WHZ ≥−2.0 at admission, and 73 % presented severe malnutrition (WHZ <−3). When using local references, 56% of children had a WHZ ≥−2.0 at admission, and only 8% presented severe malnutrition. Of course, malnutrition is better defined using NCHS references than local ones in a developing country such as Burkina Faso. But both references were used to standardise nutritional status in our study because age and sex reference values for IGF-I were available at the local level only.

We observed variations of IGF-I with respect to the nutritional rehabilitation. Low IGF-I values were observed in hospital children suffering from protein and energy malnutrition [22, 23]. But values were lower in children suffering from marasmus than in children suffering from kwashiorkor; this suggests that the secretion of IGF-I is closely related to energy consumption than to protein consumption [22]. After the nutritional rehabilitation of children admitted for protein and energy malnutrition, the IGF-I increased significantly in children that had kwashiorkor or marasmus. But the IGF-I values after 14 days of nutritional rehabilitation were still lower than in healthy children [22, 23]. There was no good agreement between the IGF-I values and the weight for height Z-scores upon admission, owing to the fact that a severe malnutrition was present in 73% of children and IGF-I values were too low to allow for correlation. But after 14 days, we observed good agreement between IGF-I values and WHZ values; the increase in IGF-I also paralleled the increase in weight for age Z-scores. These results suggest that IGF-I can be a dynamic indicator of nutritional recovery.

The study conducted in Gabon by Zamboni et al. [22] reported IGF-I values of 2.7 ± 0.7 nmol/L upon admission of 9 children (mean age: 17.2 ± 5.4 months) with kwashiorkor and the IGF-I increased to 7.6 ± 1.7 nmol/L after 4–8 weeks of nutritional rehabilitation. In children with marasmus (*n* = 13), the values of IGF-I were 1.6 ± 0.4 nmol/L on admission and 5.2 ± 1.1 nmol/L after complete nutritional rehabilitation [22].

Palacio et al. [24] found that IGF-I concentrations increased significantly after 10% weight recovery in malnourished children (*n* = 15). On admission, the IGF-I values were 3.5 ± 1.0 ng/mL and reached 5.9 ± 2.0 ng/mL after 10% body weight gain [22].

Bhutta et al. [23] and Palacio et al. [24] reported that an increase in IGF-I rates in malnourished children and its relation with weight gain was observed during the rehabilitation. This was not observed for markers such as prealbumin or serum albumin [23, 24].

Smith et al. [29] observed that the caloric restriction induced a reduction in IGF-I and IGFBP-3 in adults and children. After nutritional rehabilitation, an increase was achieved, but without reaching the initial rates. A reduction in IGF-I rates was also reported in other types of malnutrition such as mental anorexia, coeliac, acquired immune deficiency syndrome (AIDS), or Crohn's disease [30, 31].

The ability of IGF-I to follow variations of nutritional status shows that it is potentially a good clinical marker to follow nutritional rehabilitation in children with protein and energy malnutrition. The prognostic interest of IGF-I remains to be demonstrated by the implementation of studies which will measure the impact of IGF-I on the mortality of children with malnutrition in comparison with other markers of nutrition like albumin, prealbumin, and RBP. If the prognostic interest of IGF-I were proved, the use of IGF-I will be recommended. The experience shows that the cost of technology could be reduced quickly with the development of the research.

## Figures and Tables

**Table 1:** Social and demographic characteristics of the household and the children admitted in two the nutritional rehabilitation centres in Burkina Faso.

Social and demographic characteristics	N	Mean ± SD or %
Gender of the children		
Boys	37	62.7
Girls	22	37.3
Mean age of children (months)	59	14 ± 6
Age categories of children (months)		
0–5	4	6.8
6–11	6	10.2
12–23	45	76.3
24–35	4	6.8
*Age of the mothers*	56	27.3 ± 6.1
*Age categories (years)*		
≤19	6	10.7
20–24	13	23.2
25–29	15	26.8
30–34	14	25.0
35–39	8	14.3
*Scholarship of the mother*		
Yes	2	3.4
No	57	96.6
*Scholarship of the father*		
Yes	7	11.9
No	52	88.1
*Employment of the mother*		
Housewife	55	93.2
Independents/employees	4	6.8
*Employment of the father*		
Farmer	52	88.1
Independent/employees	7	11.9

**Table 2:** Weight for height and IGF-I increase during nutritional rehabilitation, in 59 malnourished children in Burkina Faso.

Absolute measurements		*Weight* (kg)		**IGF-I** ratio to admission
	*n*	Mean ± SD	*n*	[95% confidence interval]
Admission	59	5.520 ± 1.003	59	1
After 7 days	59	5.943 ± 1.022	59	1.59 [1.29; 1.89]
After 14 days	59	6.362 ± 1.143	59	2.52 [2.10; 2.94]
Age- and sex-adjusted* weight for height Z-score				
	*n*	Mean ± SD		
Admission	59	−3.56 ± 0.85		
After 7 days	59	−3.05 ± 0.79		
After 14 days	59	−2.44 ± 1.06		
Age- and sex-adjusted** weight for height Z-score				**IGF-I** Z-score
	*n*	Mean ± SD	*n*	Mean ± SD
Admission	59	−1.87 ± 0.89	59	−2.55 ± 0.80
After 7 days	59	−1.45 ± 0.90	59	−2.03 ± 1.22
After 14 days	59	−1.07 ± 1.02	59	−1.16 ± 1.39

Reference values from Burkina Faso.

**International reference values.
